# Xenogeneic human umbilical cord-derived mesenchymal stem cells reduce mortality in rats with acute respiratory distress syndrome complicated by sepsis

**DOI:** 10.18632/oncotarget.17320

**Published:** 2017-04-21

**Authors:** Fan-Yen Lee, Kuan-Hung Chen, Christopher Glenn Wallace, Pei-Hsun Sung, Jiunn-Jye Sheu, Sheng-Ying Chung, Yung-Lung Chen, Hung-I Lu, Sheung-Fat Ko, Cheuk-Kwan Sun, Hsin-Ju Chiang, Hsueh-Wen Chang, Mel S. Lee, Hon-Kan Yip

**Affiliations:** ^1^ Division of Thoracic and Cardiovascular Surgery, Department of Surgery, Kaohsiung Chang Gung Memorial Hospital and Chang Gung University College of Medicine, Kaohsiung, Taiwan; ^2^ Department of Anesthesiology, Kaohsiung Chang Gung Memorial Hospital and Chang Gung University College of Medicine, Kaohsiung, Taiwan; ^3^ Department of Plastic Surgery, Royal Devon & Exeter Hospital, Exeter, United Kingdom; ^4^ Division of Cardiology, Department of Internal Medicine, Kaohsiung Chang Gung Memorial Hospital and Chang Gung University College of Medicine, Kaohsiung, Taiwan; ^5^ Department of Radiology, Kaohsiung Chang Gung Memorial Hospital and Chang Gung University College of Medicine, Kaohsiung, Taiwan; ^6^ Department of Emergency Medicine, E-Da Hospital, I-Shou University School of Medicine for International Students, Kaohsiung, Taiwan; ^7^ Department of Obstetrics and Gynecology, Kaohsiung Chang Gung Memorial Hospital and Chang Gung University College of Medicine, Kaohsiung, Taiwan; ^8^ Department of Biological Sciences, National Sun Yat-Sen University, Kaohsiung, Taiwan; ^9^ Department of Orthopedics, Kaohsiung Chang Gung Memorial Hospital and Chang Gung University College of Medicine, Kaohsiung, Taiwan; ^10^ Institute for Translational Research in Biomedicine, Kaohsiung Chang Gung Memorial Hospital and Chang Gung University College of Medicine, Kaohsiung, Taiwan; ^11^ Center for Shockwave Medicine and Tissue Engineering, Kaohsiung Chang Gung Memorial Hospital and Chang Gung University College of Medicine, Kaohsiung, Taiwan; ^12^ Department of Nursing, Asia University, Taichung, Taiwan; ^13^ Department of Medical Research, China Medical University Hospital, China Medical University, Taichung, Taiwan

**Keywords:** acute respiratory distress syndrome, sepsis syndrome, inflammatory and immune reactions, xenogeneic mesenchymal stem cell, mortality

## Abstract

This study tested the hypothesis that xenogeneic human umbilical cord-derived mesenchymal stem cell (HUCDMSC) therapy would improve survival rates in rats with acute respiratory distress-syndrome (ARDS, induction by 48 h inhalation of 100% oxygen) and sepsis-syndrome (SS, induction by cecal-ligation and puncture) (ARDS-SS). Adult-male Sprague-Dawley rats were categorized into group 1 (sham-controls), group 2 (ARDS-SS), group 3 [ARDS-SS+HUCDMSC (1.2 ×10^6^ cells administered 1 h after SS-induction)], and group 4 [ARDS-SS+HUCDMSC (1.2 ×10^6^ cells administered 24 h after SS-induction)]. The mortality rate was higher in groups 2 and 4 than in groups 1 and 3 (all p<0.0001). The blood pressure after 28 h was lower in groups 2, 3 and 4 (p<0.0001) than in group 1. Albumin levels and percentages of inflammatory cells in broncho-alveolar lavage fluid, and the percentages of inflammatory and immune cells in circulation, were lowest in group 1, highest in group 2, and higher in group 3 than group 4 (all p<0.0001). The percentages of inflammatory cells in ascites and kidney parenchyma showed identical patterns, as did kidney injury scores (all p<0.0001). EarlyHUCDMSC therapy reduced rodent mortality after induced ARDS-SS.

## INTRODUCTION

Acute respiratory distress syndrome (ARDS) and sepsis syndrome with multiple organ failure (SS) are two global, growing diseases that have high in-hospital mortality [1–5]. The in-hospital mortality rate has been estimated to be from 26% to 63% for ARDS patients [6–8], and 60% to 80% for SS patients [9–12].

The innate and adaptive immune responses defend the body from non-infectious, harmful substances and invasive microorganisms. Patients with both ARDS and SS (ARDS-SS) experience an uncontrolled immune response, inflammation, over-production of reactive oxygen species (ROS), over-activation (i.e., cascade) of complement, and endotoxin release from infectious microorganisms [4, 10, 13–24]. These complex hyper-inflammatory and immune responses are triggered in an attempt to eliminate the causative factors, but they also cause multi-organ damage in the host [17, 20], which could contribute to the high mortality in ARDS-SS [13–24]. Strategies that target these increased inflammatory and immune responses may have therapeutic potential.

Mesenchymal stem cell (MSC) therapy has the capacity to attenuate inflammation [16, 25–28] and down-regulate innate and adaptive immunity [16, 26–30] through suppressing immunogenicity [16, 26–31]. MSC therapy might improve the clinical outcomes in ARDS-SS patients. However, prior to apply MSC therapy for human being, a preclinical study had to be first performed by using human being derived MSC (i.e., xenogeneic MSC) to prove not only the safety and efficacy but also the immune privilege of the MSC regardless for what kind of biological species. Accordingly, this preclinical study tested the hypothesis that xenogeneic human umbilical cord-derived mesenchymal stem cell (HUCDMSC) therapy could safely and effectively protect rodents from ARDS-SS, and improve prognostic outcome.

## RESULTS

### The albumin and inflammatory cytokine levels of bronchoalveolar lavage (BAL) fluid by day 5 after ARDS-SS induction

**Figure 1:**
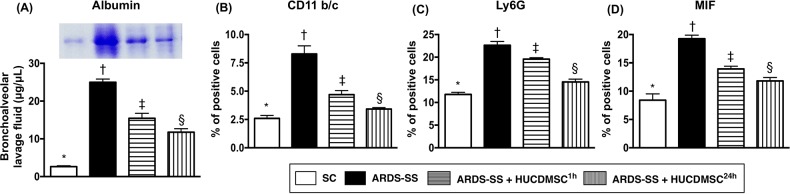
Albumin and proinflammatory cytokine levels in BAL fluid (**A**) The albumin level in bronchoalveolar lavage (BAL) fluid, * vs. other groups with different symbols (†, ‡, §), p<0.001. (**B**) Flow cytometric analysis of CD11b/c+ cells in BAL fluid, * vs. other groups with different symbols (†, ‡, §), p<0.0001. (**C**) Flow cytometric analysis of Ly6G+ cells in BAL fluid, * vs. other groups with different symbols (†, ‡, §), p<0.0001. (**D**) Flow cytometric analysis of macrophage migratory inhibitor factor (MIF)-1+ cells in BAL fluid, * vs. other groups with different symbols (†, ‡, §), p<0.0001. Statistical analyses were performed by one-way ANOVA, followed by Bonferroni multiple comparison post hoc test (n=6 for each group). Symbols (*, †, ‡, §) indicate significance at the 0.05 level. SC = sham control; ARDS = acute respiratory distress syndrome; SS = sepsis syndrome; HUCDMSC = human umbilical cord-derived mesenchymal stem cell; ^1h^ indicated HUCDMSC administration at 1 hour after SS induction; ^24h^ indicated HUCDMSC administration at 24 hour after SS induction.

BAL albumin level was lowest in group 1 (SC) and highest in group 2 (ARDS-SS). Group 3 (ARDS-SS + HUCDMSC^1h^) showed a higher BAL albumin level than in group 4 (ARDS-SS + HUCDMSC^24h^) (Figure 1). Flow cytometry showed the same pattern between the four groups for three inflammatory biomarkers: CD11b/c, macrophage migration inhibitor factor (MIF), and Ly6G.

### Blood pressure and mortality by day 5 after ARDS-SS induction

**Figure 2:**
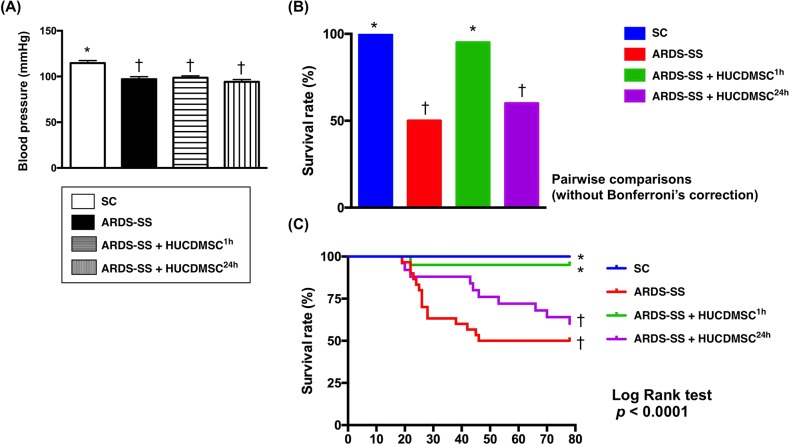
Systolic blood pressure (SBP) at 28 h after sepsis induction and mortality rate by day 5 after ARDS-SS induction (**A**) SBP was measured from rat tail at 28 h after sepsis syndrome induction, * vs. †, p<0.001. **(B)** The result of Pair comparisons (i.e., expressed as bar charts) showed that the 5-day survival rate was higher in sham control and ARDS-SS + HUCDMSC^1h^ than in ARDS-SS + HUCDMSC^24h^. † vs. ‡, p<0.0003. **(C)** Kaplan Meier curve showed 5-day cumulative survival rate among the four groups. * vs. other groups with different symbols (†, ‡), p<0.0001 (for 4 groups by log rank test). Symbols (*, †, ‡) indicate significance at the 0.05 level. SC = sham control; ARDS = acute respiratory distress syndrome; SS = sepsis syndrome; HUCDMSC = human umbilical cord-derived mesenchymal stem cell; ^1h^ indicated HUCDMSC administration at 1 hour after SS induction; ^24h^ indicated HUCDMSC administration at 24 hour after SS induction.

By 28 h after sepsis induction, the systolic blood pressure was higher in group 1 than groups 2, 3, and 4, with no difference among groups 2, 3, and 4 (Figure 2). The mortality rate was higher in groups 2 and 4 (50%, 40%, respectively) than in groups 1 and 3 (0%, 5%, respectively), P <0.0001 for 4 groups by log rank test. Additionally, pairwise comparisons (without Bonferroni's correction) showed that: (1) Group 1 vs. 2, p = 0.0002; (2) Group 1 vs. 3, p = 0.317; (3) Group 1 vs. 4, p = 0.002; (4) Group 2 vs. 3, P = 0.002; (5) Group 2 vs. 4, p = 0.306; (6) Group 3 vs. 4: p = 0.009 (Figure 2). We noted that the majority of animals in group 4 (ARDS-SS + HUCDMSC^24h^) died after the HUCDMSCs were administered. This suggests only prompt HUCDMSC treatment lowered mortality after ARDS-SS induction.

### Kidney injury and WT-1 expression by day 5 after ARDS-SS induction

**Figure 3:**
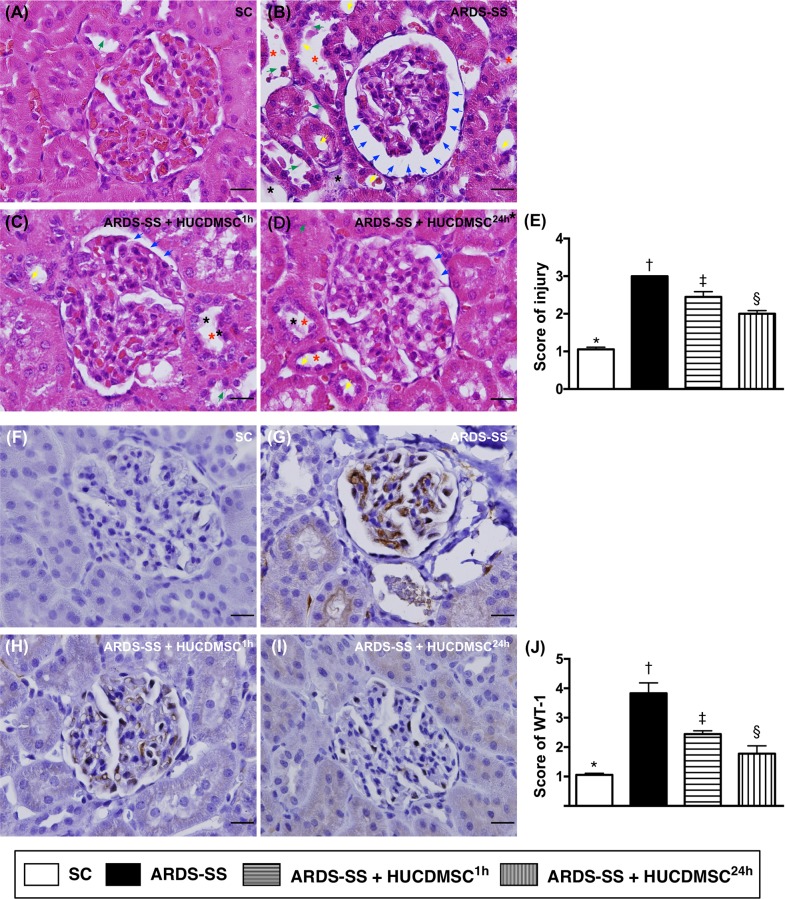
Histopathological findings of rat kidney injury and cellular expression of WT-1 by day 5 after ARDS-SS induction (**A to D**) Light microscopic findings of H & E stain (400x), demonstrating higher degree of loss of brush border in renal tubules (yellow arrows), tubular necrosis (green arrows), tubular dilation (red asterisk), protein cast formation (black asterisk), and dilation of Bowman's capsule (blue arrows) in ARDS-SS group than in other groups. (**E**) * vs. other groups with different symbols (†, ‡, §), p<0.0001. (**F to I**) Illustrating the microscopic finding (400x) of immunohistochemical staining for identification of Wilm's tumor suppressor gene 1 (WT-1), expressed predominantly in podocytes (gray color). **(J)** Analytical results of WT-1 expression, * vs. other groups with different symbols (†, ‡, §), p<0.0001. Scale bars: 20 μm. All statistical analyses were performed by one-way ANOVA, followed by Bonferroni multiple comparison post hoc test (n=8 for each group). Symbols (*, †, ‡, §) indicate significance at 0.05 level. SC = sham control; ARDS = acute respiratory distress syndrome; SS = sepsis syndrome; HUCDMSC = human umbilical cord-derived mesenchymal stem cell; ^1h^ indicated HUCDMSC administration at 1 hour after SS induction; ^24h^ indicated HUCDMSC administration at 24 hour after SS induction.

Kidney assessment was necessary because it is the major vulnerable abdominal visceral organ to be involved in cecal ligation and puncture (CLP)-induced SS. Light microscopy of hematoxylin and eosin (H & E)-stained kidney sections showed that average kidney injury score was highest in group 2, lowest in group 1, and higher in group 3 than in group 4. Immunohistochemical (IHC) staining demonstrated an identical pattern in the expression of Wilm's tumor suppressor gene (WT-1), predominantly in podocytes (Figure 3).

### Inflammatory cells in parenchyma of the kidney by IF by day 5 after ARDS-SS induction

**Figure 4:**
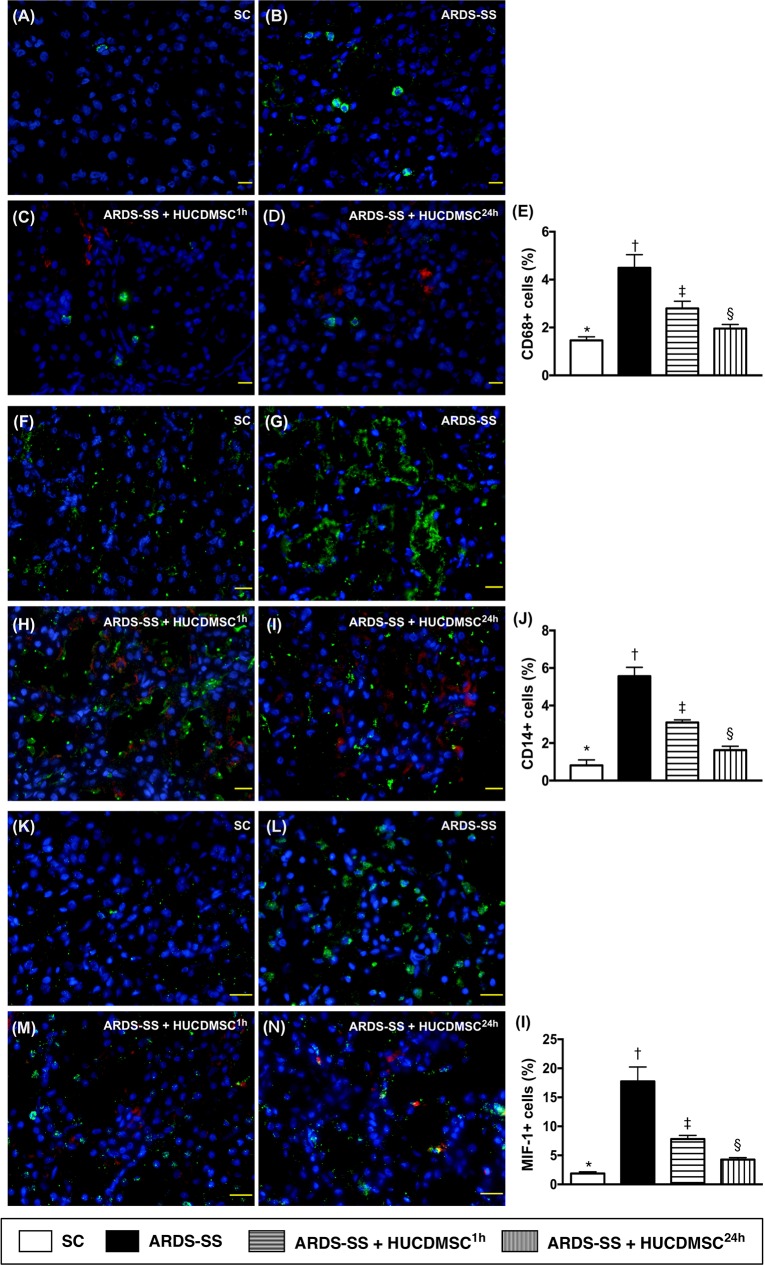
Inflammatory cell infiltration in kidney parenchyma by day 5 after ARDS-SS induction (**A to D**) Immunofluorescent (IF) microscopic finding (400x) for identification of CD68+ cells (green color). Nuclei were stained by DAPI (blue color). Red color in (C) and (D) indicated HUCDMSCs were found in kidney parenchyma. (**E**) Analytical result of number of CD68+ cells among the four groups, * vs. other groups with different symbols (†, ‡, §), p<0.0001. (**F to I**) IF microscopy (400x) for identification of CD14+ cells (green color). Nuclei were stained by DAPI (blue color). Red color in (H) and (I) indicated HUCDMSCs were found in kidney parenchyma. (**J**) Analytical result of number of CD14+ cells among the four groups, * vs. other groups with different symbols (†, ‡, §), p<0.0001. (**K to M**) Immunofluorescent (IF) microscopy (400x) for identification of macrophage migratory inhibitor factor (MIF)+ cells (green color). Nuclei were stained by DAPI (blue color). Red color in (L) and (M) indicated HUCDMSCs were found in kidney parenchyma. (**N**) Analytical result of number of MIF+ cell among the four groups, * vs. other groups with different symbols (†, ‡, §), p<0.0001. Scale bars: 20 μm. All statistical analyses were performed by one-way ANOVA, followed by Bonferroni multiple comparison post hoc test (n=8 for each group). Symbols (*, †, ‡, §) indicate significance (at 0.05 level). SC = sham control; ARDS = acute respiratory distress syndrome; SS = sepsis syndrome; HUCDMSC = human umbilical cord-derived mesenchymal stem cell; ^1h^ indicated HUCDMSC administration at 1 hour after SS induction; ^24h^ indicated HUCDMSC administration at 24 hour after SS induction.

Immunofluorescent (IF) microscopy found that the percentages of cellular expression of three inflammatory biomarkers (CD68, CD14 and MIF) were highest in group 2, lowest in group 1, and higher in group 3 than in group 4 (Figure 4).

### Kidney injury biomarker and podocyte levels by day 5 after ARDS-SS induction

**Figure 5:**
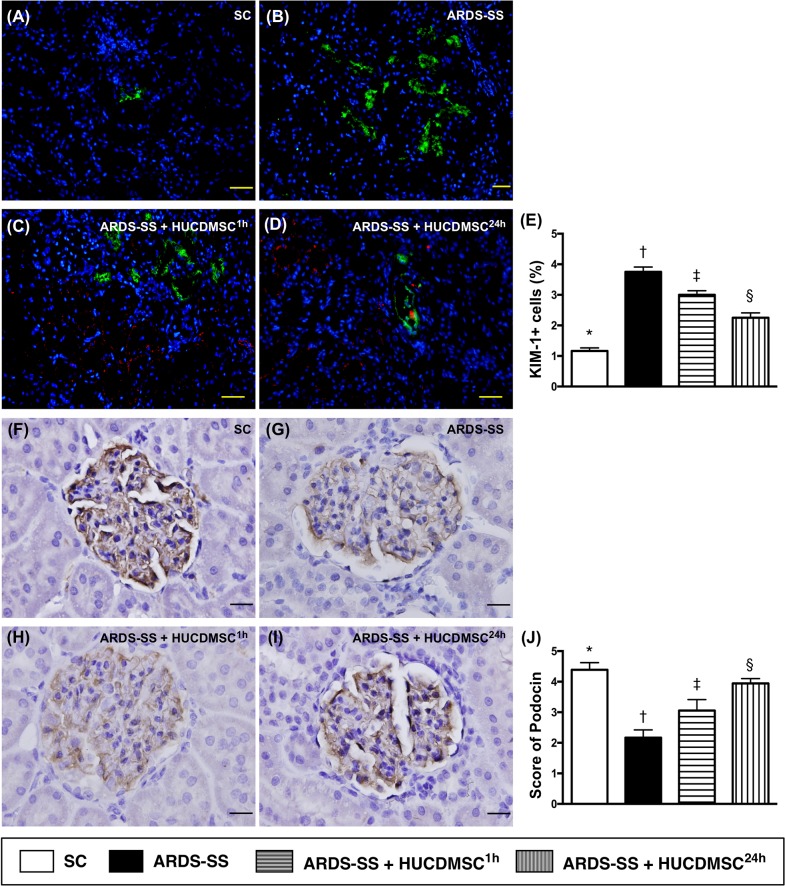
Microscopy of kidney injury biomarker and podocyte component in kidney parenchyma by day 5 after ARDS-SS induction (**A to D**) Immunofluorescent (IF) staining (400x) of kidney injury molecule (KIM)-1+ cells (green color). Nuclei were stained by DAPI (blue color). Red color in (C) and (D) indicated HUCDMSCs were found in kidney parenchyma. **(E)** Analytical result of number of KIM-1+ cells among the four groups * vs. other groups with different symbols (†, ‡, §), p<0.0001. **(F to I)** Immunohistochemical (IHC) microscopy (400x) for identification of cellular expression of podocin (gray color). **(J)** Analytical result of number of KIM-1+ cells among the four groups * vs. other groups with different symbols (†, ‡, §), p<0.0001. Scale bars: 20 μm. All statistical analyses were performed by one-way ANOVA, followed by Bonferroni multiple comparison post hoc test (n=8 for each group). Symbols (*, †, ‡, §) indicate significance at 0.05 level. SC = sham control; ARDS = acute respiratory distress syndrome; SS = sepsis syndrome; HUCDMSC = human umbilical cord-derived mesenchymal stem cell; ^1h^ indicated HUCDMSC administration at 1 hour after SS induction; ^24h^ indicated HUCDMSC administration at 24 hour after SS induction.

IF microscopy showed that the cellular expression of kidney injury molecule (KIM)-1, a kidney injury biomarker predominant in renal tubules, was increased the most in group 2. KIM-1 was higher in groups 3 and 4 than in group 1, and higher in group 3 than group 4. IHC microscopy showed that the cellular expression of podocin, one component of the podocyte foot process, did not match the KIM-1 pattern between the four groups (Figure 5).

### Protein expression of inflammation biomarkers in parenchyma of the kidney by day 5 after ARDS-SS induction

**Figure 6:**
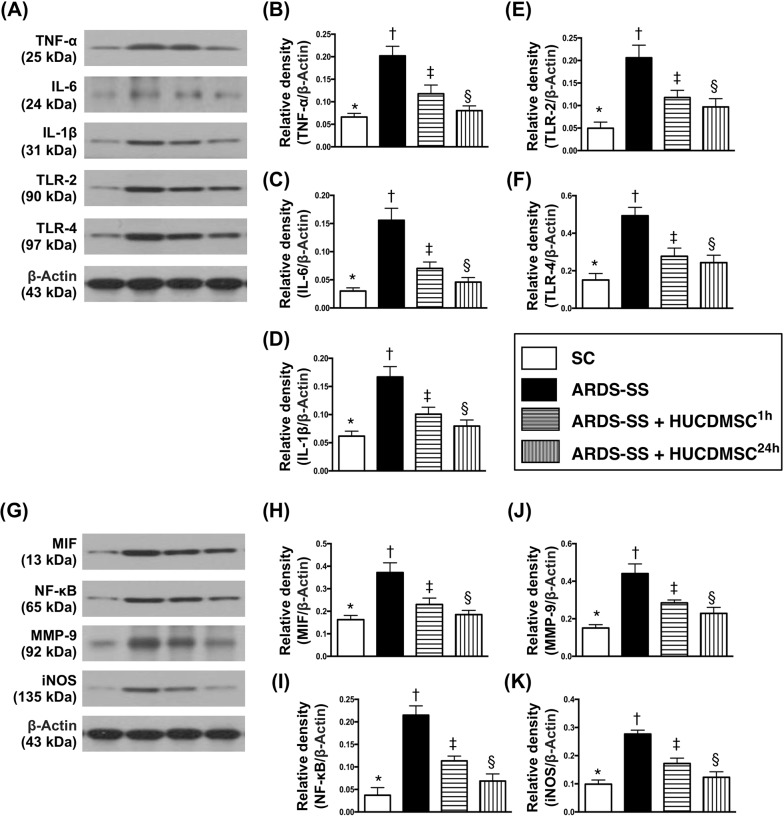
Protein expression of inflammatory biomarkers in kidney parenchyma by day 5 after ARDS-SS induction (**A**) Illustration of grouping of the protein expressions (Part I) with same actin as internal control. (**B**) Protein expression of tumor necrosis factor (TNF)-α, * vs. other groups with different symbols (†, ‡, §), p<0.0001. (**C**) Protein expression of interleukin (IL)-6, * vs. other groups with different symbols (†, ‡, §), p<0.0001. (**D**) Protein expression of IL-1Δ, * vs. other groups with different symbols (†, ‡, §), p<0.001. (**E**) Protein expression of tall-like receptor (TLR)-2, * vs. other groups with different symbols (†, ‡, §), p<0.0001. (**F**) Protein expression of TLR-4, * vs. other groups with different symbols (†, ‡, §), p<0.0001. (**G**) Illustrating grouping of the protein expressions (Part II) with same actin as internal control. (**H**) Protein expression of macrophage migratory inhibitor factor (MIF), * vs. other groups with different symbols (†, ‡, §), p<0.001. (**I**) Protein expression of nuclear factor (NF)-κB, * vs. other groups with different symbols (†, ‡, §), p<0.0001. (**J**) Protein expression of matrix metalloproteinase (MMP)-9, * vs. other groups with different symbols (†, ‡, §), p<0.001. (**K**) Protein expression of inducible nitric oxide synthase (iNOS), * vs. other groups with different symbols (†, ‡, §), p<0.001. All statistical analyses were performed by one-way ANOVA, followed by Bonferroni multiple comparison post hoc test (n=8 for each group). Symbols (*, †, ‡, §) indicate significance at 0.05 level. SC = sham control; ARDS = acute respiratory distress syndrome; SS = sepsis syndrome; HUCDMSC = human umbilical cord-derived mesenchymal stem cell; ^1h^ indicated HUCDMSC administration at 1 hour after SS induction; ^24h^ indicated HUCDMSC administration at 24 hour after SS induction.

The protein levels of nine indicators of inflammation [tumor necrosis factor (TNF)-α, interleukin (IL)-6, IL-1Δ, MIF, nuclear factor (NF)-κB, matrix metalloproteinase (MMP)-9, inducible nitric oxide synthase (iNOS), tall-like receptor (TLR)-2 and TLR-4] were highest in group 2. The protein levels were also higher in group 3 than in groups 1 and 4, and higher in group 4 than in group 1 (Figure 6).

### Protein expression of oxidative stress and apoptotic biomarkers in parenchyma of the kidney by day 5 after ARDS-SS induction

**Figure 7:**
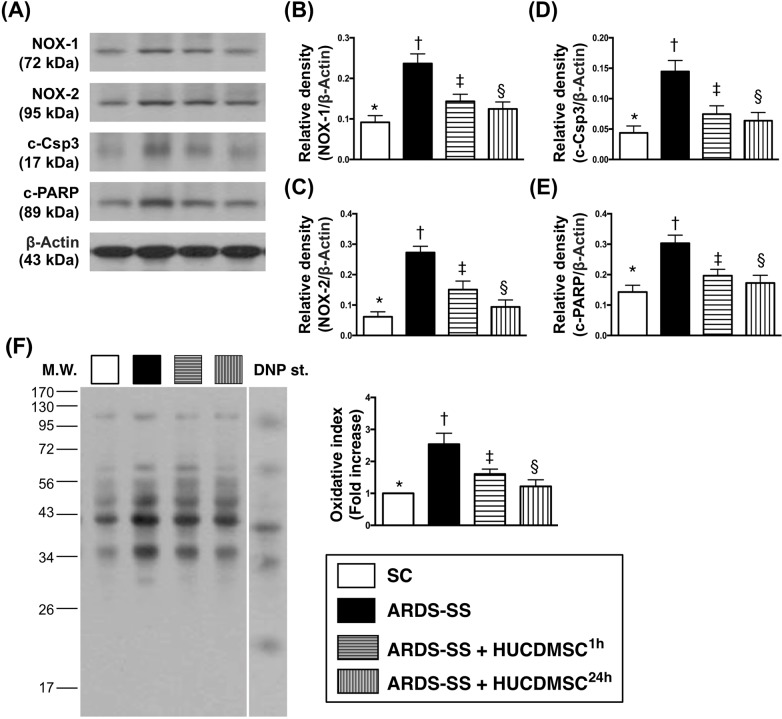
Protein expression of oxidative stress and apoptotic biomarkers in kidney parenchyma by day 5 after ARDS-SS induction (**A**) Illustration of grouping of the protein expressions (Part I) with same actin as internal control. (**B**) Protein expression of NOX-1, * vs. other groups with different symbols (†, ‡, §), p<0.001. (**C**) Protein expression of NOX-2, * vs. other groups with different symbols (†, ‡, §), p<0.0001. (**D**) Protein expression of cleaved caspase 3 (c-Casp3), * vs. other groups with different symbols (†, ‡, §), p<0.001. **(E)** Protein expression of cleaved poly (ADP-ribose) polymerase (c-PARP), * vs. other groups with different symbols (†, ‡, §), p<0.0001. **(F)** Oxidized protein expression, * vs. other groups with different symbols (†, ‡, §), p<0.001. (Note: left and right lanes shown on the upper panel represent protein molecular weight marker and control oxidized molecular protein standard, respectively). M.W = molecular weight; DNP = 1-3 dinitrophenylhydrazone. All statistical analyses were performed by one-way ANOVA, followed by Bonferroni multiple comparison post hoc test (n=8 for each group). Symbols (*, †, ‡, §) indicate significance at 0.05 level. SC = sham control; ARDS = acute respiratory distress syndrome; SS = sepsis syndrome; HUCDMSC = human umbilical cord-derived mesenchymal stem cell; ^1h^ indicated HUCDMSC administration at 1 hour after SS induction; ^24h^ indicated HUCDMSC administration at 24 hour after SS induction.

The protein levels of three indicators of oxidative stress (NOX-1, NOX-2, and oxidized protein) were highest in group 2, lowest in group 1, and higher in group 3 than in group 4. The same pattern between the four groups was observed in the protein expression of two indicators of apoptosis [cleaved caspase 3 and cleaved poly (ADP-ribose) polymerase (PARP)] (Figure 7).

### Inflammation biomarkers in ascites and circulation by flow cytometry by day 5 after ARDS-SS induction

**Figure 8:**
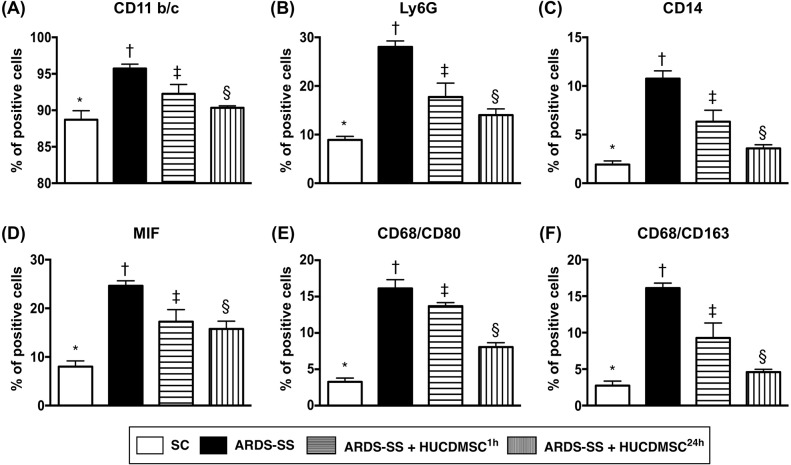
Flow cytometry analysis of inflammatory biomarkers in ascites by day 5 after ARDS-SS induction (**A**) Flow cytometry analysis of CD11b/c+ cells in ascites, * vs. other groups with different symbols (†, ‡, §), p<0.001. (**B**) Flow cytometry analysis of Ly6G+ cells in ascites, * vs. other groups with different symbols (†, ‡, §), p<0.0001. (**C**) Flow cytometry analysis of CD14+ cells in ascites, * vs. other groups with different symbols (†, ‡, §), p<0.0001. (**D**) Flow cytometry analysis of macrophage migratory inhibitor (MIF)+ cells in ascites, * vs. other groups with different symbols (†, ‡, §), p<0.0001. (**E**) Flow cytometry analysis of CD68/CD80+ cells in ascites, * vs. other groups with different symbols (†, ‡, §), p<0.0001. (**F**) Flow cytometry analysis of number of CD68/CD163+ cells in ascites, * vs. other groups with different symbols (†, ‡, §), p<0.001. All statistical analyses were performed by one-way ANOVA, followed by Bonferroni multiple comparison post hoc test (n=8 for each group). Symbols (*, †, ‡, §) indicate significance at 0.05 leve). SC = sham control; ARDS = acute respiratory distress syndrome; SS = sepsis syndrome; HUCDMSC = human umbilical cord-derived mesenchymal stem cell; ^1h^ indicated HUCDMSC administration at 1 hour after SS induction; ^24h^ indicated HUCDMSC administration at 24 hour after SS induction.

**Figure 9:**
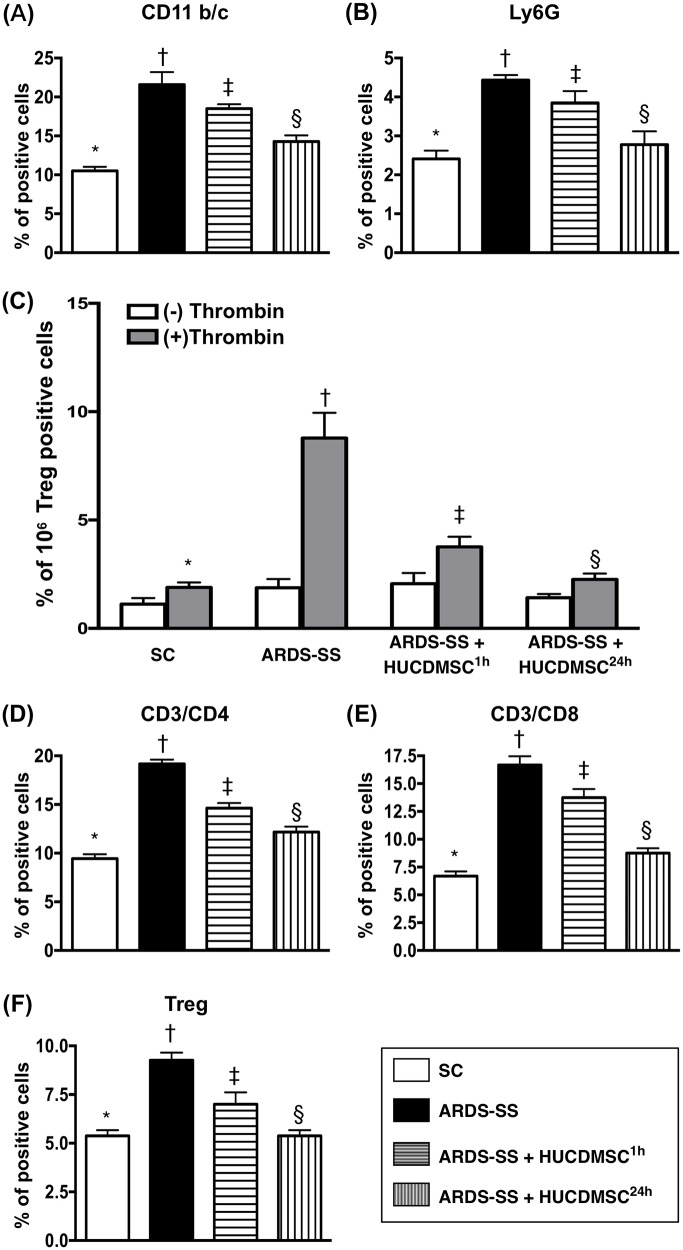
Flow cytometry analysis of inflammatory and immune cells in circulation by day 5 after ARDS-SS induction (**A**) Flow cytometry analysis of CD11b/c+ cells in circulation, * vs. other groups with different symbols (†, ‡, §), p<0.0001. (**B**) Flow cytometry analysis of Ly6G+ cells in circulation, * vs. other groups with different symbols (†, ‡, §), p<0.0001. (**C**) Flow cytometry analysis of VCAM-1+ cells in circulating platelets, * vs. other groups with different symbols (†, ‡, §), p<0.0001. (−) and (+) indicated without and with thrombin stimulation. (**D**) Flow cytometry analysis of CD3/CD4+ cells in circulation, * vs. other groups with different symbols (†, ‡, §), p<0.0001. (**E**) Flow cytometry analysis of CD3/CD8+ cells in circulation, * vs. other groups with different symbols (†, ‡, §), p<0.0001. (**F**) Flow cytometry analysis of Treg+ cells in circulation, * vs. other groups with different symbols (†, ‡, §), p<0.0001. All statistical analyses were performed by one-way ANOVA, followed by Bonferroni multiple comparison post hoc test (n=8 for each group). Symbols (*, †, ‡, §) indicate significance at 0.05 level. SC = sham control; ARDS = acute respiratory distress syndrome; SS = sepsis syndrome; HUCDMSC = human umbilical cord-derived mesenchymal stem cell; ^1h^ indicated HUCDMSC administration at 1 hour after SS induction; ^24h^ indicated HUCDMSC administration at 24 hour after SS induction.

In ascites, flow cytometry demonstrated that the percentages of cells testing positive for six inflammation biomarkers (CD11b/c, MIF, Ly6G, CD14, CD68/CD80, and CD68/CD163) were highest in group 2, lowest in group 1, and higher in group 3 than in group 4 (Figure 8). In the circulation, an identical pattern was observed of three indicators of inflammation response (CD11b/c, Ly6G, and VCAM-1) (Figure 9). Circulating levels of three indicators of immune cells (CD3/CD4, CD3/CD8, and Treg cells) also showed the same pattern among the four groups (Figure 9).

### Immunofluorescence of inflammatory cells in ascites by day 5 after ARDS-SS induction

**Figure 10:**
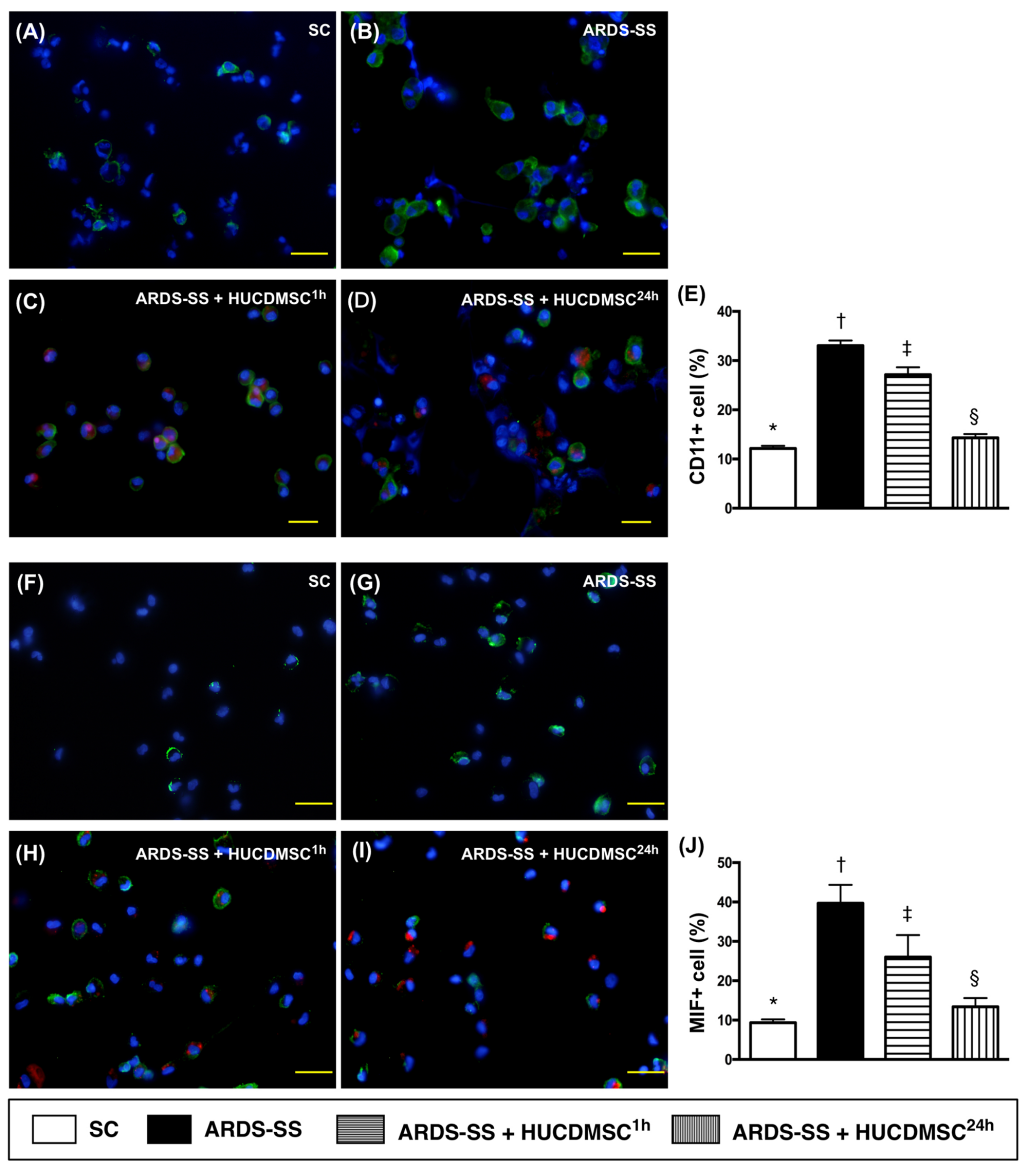
Cellular expression of CD11+ and MIF+ cells in ascites by day 5 after ARDS-SS induction (**A to D**) Immunofluorescent (IF) staining (400x) of CD11+ cells in ascites (green color). Nuclei were stained by DAPI (blue color). Red color in (C) and (D) indicated HUCDMSCs were found in kidney parenchyma. (**E**) Analytical result of CD11+ cells among the four groups, * vs. other groups with different symbols (†, ‡, §), p<0.0001. (**F to I**) IF image (400x) of macrophage migratory inhibitor (MIF)+ cells in ascites (green color). Nuclei were stained by DAPI (blue color). Red color in (H) and (I) indicated HUCDMSCs were found in kidney parenchyma. (**J**) Analytical of MIF+ cells among the four groups, * vs. other groups with different symbols (†, ‡, §), p<0.0001. Scale bars: 20 μm. All statistical analyses were performed by one-way ANOVA, followed by Bonferroni multiple comparison post hoc test (n=8 for each group). Symbols (*, †, ‡, §) indicate significance at 0.05 level. SC = sham control; ARDS = acute respiratory distress syndrome; SS = sepsis syndrome; HUCDMSC = human umbilical cord-derived mesenchymal stem cell; ^1h^ indicated HUCDMSC administration at 1 hour after SS induction; ^24h^ indicated HUCDMSC administration at 24 hour after SS induction.

**Figure 11:**
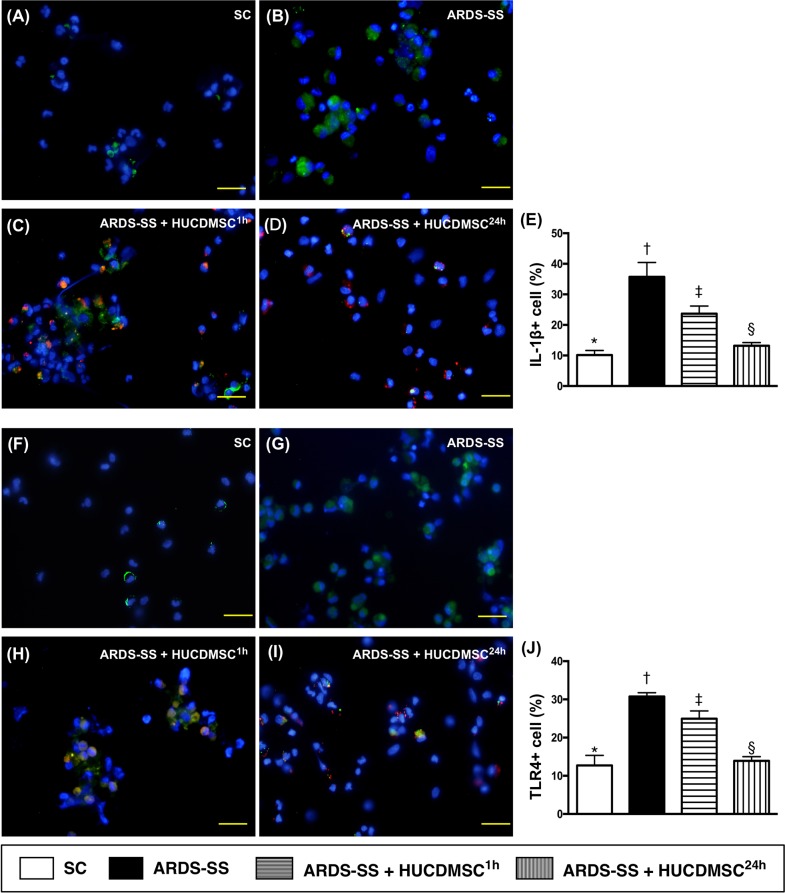
Cellular expression of IL-1Δ+ and TLR+4 cells in ascites by day 5 after ARDS-SS induction (**A to D**) Immunofluorescent (IF) image (400x) of interleukin (IL)-1Δ+ cells in ascites (green color). Nuclei were stained by DAPI (blue color). Red color in (C) and (D) indicated HUCDMSCs were found in kidney parenchyma. (**E**) Analytical result of number of IL-1Δ+ cells among the four groups, * vs. other groups with different symbols (†, ‡, §), p<0.0001. (**F to I**) IF (400x) of tall-like receptor (TLR)-4+ cells in ascites (green color). Nuclei were stained by DAPI (blue color). Red color in (H) and (I) indicated HUCDMSCs were found in kidney parenchyma. (**J**) Analytical result of TLR-4+ cells among the four groups, * vs. other groups with different symbols (†, ‡, §), p<0.0001. Scale bars: 20 μm. All statistical analyses were performed by one-way ANOVA, followed by Bonferroni multiple comparison post hoc test (n=8 for each group). Symbols (*, †, ‡, §) indicate significance at 0.05 level. SC = sham control; ARDS = acute respiratory distress syndrome; SS = sepsis syndrome; HUCDMSC = human umbilical cord-derived mesenchymal stem cell; ^1h^ indicated HUCDMSC administration at 1 hour after SS induction; ^24h^ indicated HUCDMSC administration at 24 hour after SS induction.

IF microscopy showed that the percentage of cells positive for two inflammation biomarkers in ascites (CD11 and MIF) were highest in group 2, lowest in group 1, and higher in group 3 than in group 4 (Figure 10). The same pattern between the four groups was observed for IL-1Δ, TLR-4, and CD11 (Figure 11).

## DISCUSSION

This study investigated the impact of HUCDMSC therapy on the prognostic outcome in rats after inducing ARDS-SS. Early HUCDMSC treatment (i.e., 1 h after ARDS-SS induction) reduced rat mortality rate, but late HUCDMSC treatment (i.e., 24 h after ARDS-SS induction) did not improve the survival rate. ARDS-SS induction was always followed by an elevated inflammatory reaction, while HUCDMSC treatment suppressed it.

MSC therapy has reduced rodent mortality in SS [32], and both autologous and allogenic ADMSC have the capacity of immune privilege [16, 29, 31–33]. The present study showed that in animals with ARDS-SS, early administration of xenogeneic MSC reduces the mortality rate compared to no treatment. Exogenic MSC effectively saved ARDS-SS animals, which suggests that MSC may have universal immune privilege. Despite the success of the early treatment, late administration of xenogeneic MSC was not effective for rodents with ARDS-SS.

MSC treatment can attenuate the elevated inflammatory and immune reactions that frequently occur in acute tissue/organ injury and SS [16–18, 25–28, 31–35]. Here, inflammatory biomarkers in circulation and body fluids (i.e., BAL and ascites) were higher in ARDS-SS animals than SC animals. Circulating immune cells displayed the same trend. All of these inflammatory immunogenetic biomarkers were suppressed in ARDS-SS animals after receiving early HUCDMSC treatment, and further suppressed in ARDS-SS animals after receiving late HUCDMSC treatment.

A better prognostic outcome was coupled with an increased inflammatory and immune reaction in group 3 (i.e., ARDS-SS + HUCDMSC^1h^), and vice versa in group 4 (ARDS-SS + HUCDMSC^24h^). These unexpected findings could be explained by a few reasons. First, 95% of group 3 animals were alive at the time of treatment, and about half had severe sepsis. The resulting organ damage (i.e., kidney parenchymal damage) can lead to relatively higher inflammatory and immune reactions. Second, nearly half of the group 4 animals were dead at the late treatment, suggesting the organ damage (i.e., the kidney parenchymal damage) should be relatively less severe in the animals that survived. This less severe damage would most likely not cause an overwhelming immune response relative to the other groups. Third, half-life and functionality of HUCDMSCs in animals is a possible factor resulting in differing inflammatory responses between group 3 and group 4.

### Limitations

Although the short-term outcome in ARDS-SS rats was improved after the early HUCDMSC treatment, the long-term outcome was not studied. Despite the albumin level in BAL fluid was found to be increased in ARDS-SS, we did not study whether ARDS, SS, or ARDS-SS was the most important contributor to this increase in lung permeability.

In conclusion, early administration of xenogeneic MSC suppressed the inflammatory/immune reaction, preserved the integrity of vital organs (i.e. lung and kidney), and reduced the mortality rate in rodents with induced ARDS-SS.

## MATERIALS AND METHODS

### Ethics

All animal experiments and procedures were approved by the Institute of Animal Care and Use Committee at Kaohsiung Chang Gung Memorial Hospital (Affidavit of Approval of Animal Use Protocol No. 2011053001), and performed in accordance with the Guide for the Care and Use of Laboratory Animals [The Eighth Edition of the Guide for the Care and Use of Laboratory Animals (NRC 2011)].

Animals were housed in an Association for Assessment and Accreditation of Laboratory Animal Care International (AAALAC)-approved animal facility in our hospital with controlled temperature and light cycle (24°C and 12/12 light cycle).

### Inducing ARDS and SS in rodents

The ARDS experimental model used in this study is described in our recent studies [17, 18], wherein pure oxygen (i.e., 100% O_2_) was continuously administered to the rat for 48 h. The SS induction procedure has been described in our previous report [34], which features a cecal ligation and puncture (CLP) 48 h after ARDS induction.

The tail systolic blood pressure (SBP) was measured with a CODA monitor (Kent Scientific Corporation, U.S.A.) at 28 h after CLP by a technician who was blinded to the treatment protocols.

### Isolation and culture of HUCDMSCs

The HUCDMSCs were provided by BIONET Corp. (Taipei City, Taiwan). The protocol for HUCDMSC preparation was described as follows:

After obtaining informed consent, umbilical cords were collected and stored in DPBS (Gibco), and kept at 4°C until processing. The cords were delivered to the laboratory in a bottle of sterile saline solution, and processed within 24 h after birth. The umbilical cords were first disinfected with 75% ethanol, and then washed with PBS to remove any contaminating blood.

After removing the blood vessels to avoid endothelial cell contamination, the cord tissue was cut into small pieces (0.5-1 mm^3^). The small pieces were placed directly into 10 cm culture dishes for growth in alpha-MEM (GIBCO, USA) supplemented with 5% UltraGRO^TM^ (AventaCell, USA) and antibiotics (PSA, GIBCO). Cultures were maintained at 37°C in a humidified atmosphere containing 5% CO2, replenished with fresh medium every 3-4 days. Near confluent cultures were rinsed with DPBS, harvested with 0.05% TrypLE (GIBCO, USA), and transferred to fresh 10 cm culture dishes at a plating density of 3-6 × 10^3^ cells per cm^2^ for further growth. MSCs were then cryopreserved in culture medium containing 10% DMSO, using a control rate freezer (Icecube, Sylab, AT) and stored at -190°C in a vapor phase liquid nitrogen tank.

Umbilical cord MSCs were stained with fluorescein isothiocyanate (FITC)- or phycoerythrin (PE)- conjugated antibodies against the following surface markers for flow cytometric analysis: CD13, CD14, CD29, CD31, CD34, CD44, CD45, CD73, CD90, HLA-DR, and CD105 (BD Pharmingen, San Diego, CA). Further, all MSC batches were tested for bacterial and fungal contamination (BacT Alert, Biomérieux, USA), mycoplasma (PCR, Biological industries USA), and endotoxin (LAL single vial test, Charles River Laboratories, USA), as well as anti-HTLV, anti-HIV, RPR, HBsAg, and anti-HCV.

### Animal group treatment

Pathogen-free, adult male Sprague-Dawley (SD) rats weighing 325-350 g (Charles River Technology, BioLASCO Taiwan Co. Ltd., Taiwan) were randomized into four groups, with 16 animals per group. Group 1 was the sham control (SC) group, which was subjected to cecal exposure *without* ligation and puncture. Group 2 (ARDS-SS + saline) had 3.0 cc saline administered intra-peritoneally at 1 h after CLP. Group 3 (ARDS-SS + HUCDMSC^1h^) and group 4 (ARDS-SS + HUCDMSC^24h^) animals were intravenously administered 1.2 × 10^6^ xenogeneic cells through the penis vein at 1 h and 24 h after CLP, respectively.

For both the bronchoalveolar lavage and cellular investigations, 12 surviving animals were required in each group (n = 6 in both subgroups). Including the number of dead animals, the number of rats utilized in groups 1 to 4 were 20, 30, 20, and 25, respectively. Animals were euthanized by day 5 after ARDS-SS induction.

### Bronchoalveolar lavage, and lung specimen preparation

The preparation of lung specimens for morphometric analyses is described in our previous studies [18, 36]. To elucidate the impact of HUCDMSC treatment on suppressing the inflammatory and immune reactions in lung parenchyma after ARDS-SS induction, bronchoalveolar lavage (BAL) was performed and the BAL fluid was collected for the study in six rats from each group.

### Flow cytometric quantification of immune and inflammatory cells in circulation, ascites and BAL and abdominal ascites

The flow cytometry procedure for identification and quantification of circulating inflammatory and immune cells was based on our previous report [34]. Prior to sacrificing the animals, peripheral blood mononuclear cells (PBMCs) were obtained from the tail vein using a 27# needle. PBMCs (1.0 × 10^6^ cells) were triple-stained with FITC-anti-CD3 (BioLegend), PE-anti-CD8a (BD Bioscience, San Jose, CA, USA), and PE-Cy™5 anti-CD4 (BD Bioscience, San Jose, CA, USA). To identify CD4^+^CD25^+^Foxp3^+^ regulatory T cells (Tregs), PBMCs were triple-stained with Alexa Fluor® 488-anti-CD25 (BioLegend, San Diego, CA, USA), PE-anti-Foxp3 (BioLegend, San Diego, CA, USA), and PE-Cy™5 anti-CD4 (BD bioscience, San Jose, CA, USA) according to the manufacturer's protocol for the Foxp3 Fix/Perm buffer set. The numbers of CD3^+^CD4^+^ helper T cells, CD3^+^CD8^+^ cytotoxic T cells and CD4^+^CD25^+^Foxp3^+^ Tregs were analyzed using flow cytometry (FC500, Beckman Coulter, Brea, CA, USA).

Additionally, the numbers of inflammatory cells in circulation [i.e., CD11b/c, LyG6, vascular cell adhesion molecule (VCAM)-1], in ascites [macrophage migratory inhibitor factor (MIF), CD14, CD11b/c, LyG6, CD68/CD80, CD68/CD163], and in ABL (CD11b/c, MIF, Ly6G) were assessed using the flow cytometric method.

### Western blot analysis of kidney

The procedure and protocol for Western blot analysis were based on our recent reports [32, 35, 37]. Briefly, equal amounts (50 μg) of protein extracts were loaded and separated by SDS-PAGE using acrylamide gradients. After electrophoresis, the separated proteins were transferred electrophoretically to a polyvinylidene difluoride (PVDF) membrane (Amersham Biosciences, Amersham, UK). Nonspecific sites were blocked by incubation of the membrane in blocking buffer [5% nonfat dry milk in T-TBS (TBS containing 0.05% Tween 20)] overnight. The membranes were incubated with the following primary antibodies for 1 hour at room temperature: cleaved poly (ADP-ribose) polymerase (PARP) (1:1000, Cell Signaling, Danvers, MA, USA), cleaved caspase 3 (1:1000, Cell Signaling, Danvers, MA, USA), matrix metalloproteinase (MMP)-9 (1:3000, Abcam, Cambridge, MA, USA), tumor necrosis factor (TNF)-α (1:1000, Cell Signaling, Danvers, MA, USA), nuclear factor (NF)-κB (1:600, Abcam, Cambridge, MA, USA), macrophage migration inhibitor factor (MIF)-1 (1:2000, Abcam), tall-like receptor (TLR)-2 (1:1000, Novusbio), TLR-4 (1:500, Abcam), inducible nitric oxide synthase (iNOS) (1:200, Abcam, Cambridge, MA, USA), interleukin (IL)-1β (1:1000, Cell Signaling, Danvers, MA, USA), NOX-1 (1:2000, Sigma, St. Louis, Mo, USA), NOX-2 (1:500, Sigma, St. Louis, Mo, USA), IL-6 (1:500, Abcam), and actin (1: 10000, Chemicon, Billerica, MA, USA). Horseradish peroxidase-conjugated anti-rabbit immunoglobulin IgG (1:2000, Cell Signaling, Danvers, MA, USA) was used as a secondary antibody for one-hour incubation at room temperature. Membranes were washed eight times within one hour. Immunoreactive bands were visualized by enhanced chemiluminescence (ECL; Amersham Biosciences, Amersham, UK) and exposed to Biomax L film (Kodak, Rochester, NY, USA). For purposes of quantification, ECL signals were digitized using Labwork software (UVP, Waltham, MA, USA).

### Immunohistochemical (IHC) and immunofluorescent (IF) staining

IF staining has been described in our previous reports [32, 35, 37]. For IHC and IF staining, rehydrated paraffin sections were first treated with 3% H_2_O_2_ for 30 minutes and incubated with Immuno-Block reagent (BioSB, Santa Barbara, CA, USA) for 30 minutes at room temperature. Sections were then incubated with primary antibodies specifically against CD68 (1:100, Abcam, Cambridge, MA, USA), CD14 (1:300, BioSS, Woburn, MA, USA), kidney injury molecule (KIM)-1 (1:500, R&D Systems), MIF-1 (1:100, Abcam, Cambridge, MA, USA), and Wilm's tumor suppressor gene (WT)-1 (1:1000, Abcam, Cambridge, MA, USA). Sections incubated with irrelevant antibodies served as controls. Three sections of kidney specimens were analyzed in each rat. For quantification, three randomly selected high power fields (HPFs; 400x for IHC and IF studies) were analyzed in each section. The mean number of positively-stained cells per HPF for each animal was determined across all nine HPFs.

An IHC-based scoring system was adopted for semi-quantitative analyses of podocin and WT-1 as percentage of positive cells in a blind fashion [Score of positively-stained cell for podocin and WT-1: 0 = no stain %; 1= <15%; 2 = 15∼25%; 3 = 25∼50%; 4 = 50∼75%; 5= >75%-100%/per HPF (400 x)].

### Histopathological assessment of kidney injury at day 5 after ARDS-SS procedure

Histopathology scoring was assessed in a blinded fashion as we have previously described [32, 35, 37]. The scoring system reflecting the grading of tubular necrosis, loss of brush border, cast formation, and tubular dilatation in 10 randomly chosen, non-overlapping fields (200x) was as follows: 0 (none), 1 (≤10%), 2 (11–25%), 3 (26–45%), 4 (46–75%), and 5 (≥76%).

### Oxidative stress reaction in lung parenchyma

The procedure for assessing the protein expression of oxidative stress has been described in our previous reports [16, 18, 32, 35, 37], using the Oxyblot Oxidized Protein Detection Kit (Chemicon S7150, Billerica, MA, USA). For quantification, ECL signals were digitized using Labwork software (UVP, Waltham, MA, USA).

### Statistical analysis

Quantitative data are expressed as mean ± SD. Statistical analysis was performed by ANOVA, followed by Bonferroni multiple-comparison post hoc test. Statistical analysis was also performed using SPSS (SPSS for Windows, version 13; SPSS, IL, U.S.A.). The threshold for statistical significance was considered P < 0.05.
